# From Challenge to Opportunity: Addressing Oxidative Stress in Animal Husbandry

**DOI:** 10.3390/antiox12081543

**Published:** 2023-08-02

**Authors:** Shihai Zhang

**Affiliations:** 1 Guangdong Province Key Laboratory of Animal Nutrition Control, College of Animal Science, South China Agricultural University, Guangzhou 510642, China; zhangshihai@scau.edu.cn; 2 College of Animal Science and National Engineering Research Center for Breeding Swine Industry, South China Agricultural University, Guangzhou 510642, China; 3 Guangdong Laboratory for Lingnan Modern Agriculture, South China Agricultural University, Guangzhou 510642, China

Years of study have explored the issues caused by oxidative stress in livestock and poultry production. This stress, which is mainly the result of an imbalance between antioxidants and oxidants such as ROS and RNS, is associated with diminished feed intake, a decline in feed conversion efficiency, weakened disease immunity, and heightened mortality. Environmental factors, like inadequate management, harsh environmental stressors, lack of proper nutrition, and rough transportation, intensify this oxidative imbalance. On a microscopic scale, excessive ROS and RNS production harms proteins, lipids, and nucleic acids, unsettling the cells’ equilibrium. Additionally, disruptions in mitochondrial function boost the production of ROS/RNS, impairing energy metabolism processes. Contemporary research also underscores the complex interplay between redox signaling and the reproductive health of livestock and poultry, which is influenced by changes in gene activity and epigenetic shifts.

Identifying nutritional substances that can alleviate oxidative stress holds significant implications for livestock farming. These substances, which can range from natural compounds to specific antioxidants, have the potential to improve the overall health and productivity of livestock. This Special Issue provide novel insights into oxidative regulation and health protection in livestock and poultry. For instance, the study on Rehmannia glutinosa polysaccharides (RGP) supports its potential as a natural agent against diseases stemming from oxidation and inflammation [1]. Another compound, Chenodeoxycholic acid (CDCA), a primary bile acid, has been highlighted due to its pivotal role in regulating intestinal epithelial cell function, which is crucial for overall animal health [2]. In addition, specific amino acid mixtures were found to enhance the antioxidant status, suggesting potential strategies to boost swine industry productivity [3]. The antioxidant properties of α-ketoglutaric acid (AKG) further underscore the importance of dietary interventions in enhancing livestock health [4]. 2-hydroxy-(4-methylseleno)butanoic acid (OH-SeMet) supplementation was found to support selenoprotein expression, reduce oxidative stress, and modulate the inflammatory response, enhancing the macrophages’ phagocytic and killing abilities [5]. Traditional medicines, like Artemisia ordosica, which are recognized for their antioxidant properties, offer potential as feed additives, enhancing livestock health and resilience against oxidative stress [6]. The threat posed by mycotoxins, especially Deoxynivalenol (DON), is another area of concern. However, emerging research on histone modification in mycotoxin-induced cytotoxicity offers hope for potential interventions [7]. Polyphenols hold significant promise as feed additives for pigs; they may mitigate the oxidative stress and intestinal toxicity induced by DON [8]. Interestingly, recent research underscored the significance of redox biomarkers in assessing meat quality in lambs and kids [9]. By using these biomarkers to make advanced predictions of these issues, timely nutritional interventions can be carried out to ensure optimal meat quality and overall livestock health. 

In recent years, an increasing number of studies have shown that microbes play a role in regulating the body’s oxidative stress response. One study examined the impact of dietary compound antioxidants on finishing pigs and revealed that these antioxidants improved feed efficiency, antioxidant capacity, and meat quality. This enhancement was linked to changes in gut microbiota, specifically the modulation of colonic Peptococcus and ileal Turicibacter_sp_H121 abundance [10]. The role of probiotics, especially strains of Lactobacillus and Bifidobacteria, in addressing conditions exacerbated by oxidative stress, such as inflammatory bowel disease (IBD) and post-weaning diarrhea, has been emphasized [11]. These findings suggest that integrating probiotics into livestock diets can be a game-changer.

In conclusion, while oxidative stress undeniably poses significant challenges to livestock and poultry production, the research landscape is rich with potential solutions. From natural compounds and antioxidants to probiotics, there is a plethora of strategies waiting to be harnessed. The discovery that these measures alleviate oxidative stress in animals offers new possibilities for improving livestock and poultry production.
